# Proteomics, pathway array and signaling network-based medicine in cancer

**DOI:** 10.1186/1747-1028-4-20

**Published:** 2009-10-28

**Authors:** David Y Zhang, Fei Ye, Ling Gao, Xiaoliang Liu, Xin Zhao, Yufang Che, Hongxia Wang, Libo Wang, Josephine Wu, Dong Song, Wei Liu, Hong Xu, Bo Jiang, Weijia Zhang, Jinhua Wang, Peng Lee

**Affiliations:** 1 Department of Pathology, Mount Sinai School of Medicine, New York, NY, USA; 2 Cancer Center, First Hospital of Jilin University, Changchun, PR China; 3 Department of Pediatric Medicine, First Hospital of Jilin University, Changchun, PR China; 4 Department of Gastrointestinal Medicine, Nanfang Medical University, Guangzhou, PR China; 5 Department of Oncology, Shanghai Renji Hospital, Shanghai, PR China; 6 Department of Gastrointestinal Medicine, First Hospital of Jilin University, Changchun, PR China; 7 Department of Breast Surgery, First Hospital of Jilin University, Changchun, PR China; 8 Department of Thoracic Surgery & Bethune Chest Center, First Hospital of Jilin University, Changchun, PR China; 9 Department of Medicine, Mount Sinai School of Medicine, New York, NY, USA; 10 NYU Cancer Institute, New York University School of Medicine, New York, NY, USA; 11 Department of Pathology, New York University School of Medicine, New York, NY, USA

## Abstract

Cancer is a multifaceted disease that results from dysregulated normal cellular signaling networks caused by genetic, genomic and epigenetic alterations at cell or tissue levels. Uncovering the underlying protein signaling network changes, including cell cycle gene networks in cancer, aids in understanding the molecular mechanism of carcinogenesis and identifies the characteristic signaling network signatures unique for different cancers and specific cancer subtypes. The identified signatures can be used for cancer diagnosis, prognosis, and personalized treatment. During the past several decades, the available technology to study signaling networks has significantly evolved to include such platforms as genomic microarray (expression array, SNP array, CGH array, etc.) and proteomic analysis, which globally assesses genetic, epigenetic, and proteomic alterations in cancer. In this review, we compared Pathway Array analysis with other proteomic approaches in analyzing protein network involved in cancer and its utility serving as cancer biomarkers in diagnosis, prognosis and therapeutic target identification. With the advent of bioinformatics, constructing high complexity signaling networks is possible. As the use of signaling network-based cancer diagnosis, prognosis and treatment is anticipated in the near future, medical and scientific communities should be prepared to apply these techniques to further enhance personalized medicine.

## Introduction

### Cancer Signaling Network

Cancer is a complex disease that results from complex signaling network pathway alterations that control cell behaviors, such as proliferation and apoptosis. The complexity of signaling network is multidimensional given the exceedingly high number of components (i.e. nodes and hubs), multiple connections (i.e. edges) between pathways (i.e. cross-talk) and many feedback loops (i.e. redundancy and compensation) [1]. Furthermore, the components in each signaling network operate at different spatial and temporal scales with continuous, dynamic changes in response to cell-cell and cell-stromal interactions. This complex, dynamic signaling network collectively affects cell function and behaviors with the possibility of sub-network (or module) affecting different function or behavior. Therefore, this multidimensional complexity poses a great challenge in network biology research.

Understanding signaling networks involved in carcinogenesis significantly advances our knowledge of cancer initiation and progression, including metastasis. Signaling network alterations accumulate at each stage of carcinogenesis that results from genetic, epigenetic and environmental changes and is viewed as a multi-step model of carcinogenesis [2]. Furthermore, the specific signaling networks that reflect the hallmarks of cancer have been demonstrated and include the ability to mimic normal growth signaling, insensitivity to antigrowth signals, ability to evade apoptosis, limitless replicative potential, sustained angiogenesis, and tissue invasion and metastasis [1,3].

Signaling network research is also important in diagnosis, biomarkers, cancer progression, drug development and treatment strategies. Recently, several studies have demonstrated the feasibility of cancer signaling network-based approaches for cancer diagnosis, prognosis and therapy [4]. In this paper, we will review the latest advancements and current progress in cancer signaling network research.

### Genomic Based Approaches For Signaling Network

The ability to collect data from a large number of genes in the same sample, including gene expression and DNA alterations, opens the possibility of obtaining network-level data. Currently, the signaling network information is typically derived from genomic profiling studies including gene expression, single nucleotide polymorphism (SNP), copy number variations (CNV) and DNA methylation (see Additional file 1) [5-12]. A limitation of genomic profiling studies is that mRNA levels and DNA alterations may not accurately reflect the corresponding protein levels and fail to reveal changes in posttranscriptional protein modulation (e.g., phosphorylation, acetylation, methylation, ubiquitination, etc.) or protein degradation rates [13]. More importantly, the signaling network constructed using these approaches does not reflect the dynamic signal flow in a spatial relationship. On the other hand, the genomic changes (mRNA level, SNP, CNV, methylation) ultimately affect protein expression, activation and inactivation, which, in turn, controls cellular behavior. Therefore, the use of a proteomics approach that can add protein-protein and protein-DNA information, which more accurately reflects the signal flow and dynamic change in the signaling network and could be a valuable addition to genomic profiling studies.

### Challenges of Protein-Based Approaches

The major challenge of proteomic research is the limited assay sensitivity of analyzing cell proteins. Although each mammalian cell contains approximately 30,000 genes, the proteins coded by these genes can be as many as 200,000 to 300,000 due to alternative splicing. Furthermore, the proteins involved in cellular homeostasis, metabolism and structure are abundant and are present 10,000 to 100,000 fold greater than proteins involved in signaling networks in an individual cell. Therefore, detection and quantification of these cell signaling proteins poses a great challenge. Two dimensional gel electrophoresis (2D) or liquid chromatograph (LC) in combination with mass spectrometry (MS) is a widely used technology to identify proteins. An important advantage of these sensitive techniques is the ability to identify unknown proteins in a complex sample. However, costly instrumentation is typically required and often insufficient to detect proteins that are in low abundance.

On the other hand, antibody detection offers great sensitivity and specificity to detect known proteins in a sample. However, multiplex array, i.e. protein arrays, to identify proteins with antibodies also has limitations. For example, capture molecules are proteins themselves and tend to denature with changes in pH or temperature. Furthermore, antigen-antibody interactions are determined by complex associations between epitope sites on the target protein and the antigen-binding site on the antibody, which are both influenced by external conditions. Antibodies must exhibit strong affinities and specificity for each respective substrate, particularly when investigating the activated state of specific proteins, such as phosphorylation, glycosylation or proteolytic cleavage. Subsequently, activation-specific antibodies, routinely used in Western blots, may not be suitable in an array format, as the phosphorylation-specific site may be imbedded within the interior aspect of protein and inaccessible to the antibody.

Quantifying protein concentration represents another problem when analyzing hundreds of antibody-antigen interactions in a single array as each antibody-antigen pair possesses an independent affinity constant. However, the variation in protein concentrations in cells may be as high as 6 folds. Thus, detection methods must be developed to quantify protein concentrations over many orders of magnitude. The detection of antigen-antibody pairs is routinely performed by either sandwich assays in which two complementary antibodies to different sites of the protein are used, or by detecting a label on the protein itself. Conjugation of proteins may disturb the native folding structure of proteins and thus may destroy the antibody-antigen interaction, yielding false negatives.

### Proteomics-Based Techniques for Cancer Signaling Network Research

Although protein-based techniques such as 2D gel, MS and antibody-antigen assays have long been available, the application in clinical research is limited (see above). However, during the past decade, the technologies have significantly improved and have rapidly transitioned to the clinical laboratories. The techniques can be categorized into two groups: MS-based and antibody-based technologies (see Additional file 2). This section discusses the most commonly used protein detection techniques as well as computational methods for data analysis and network simulation.

#### Detection of unknown proteins by two dimensional gel electrophoresis and mass spectrometry

**Figure 1:**
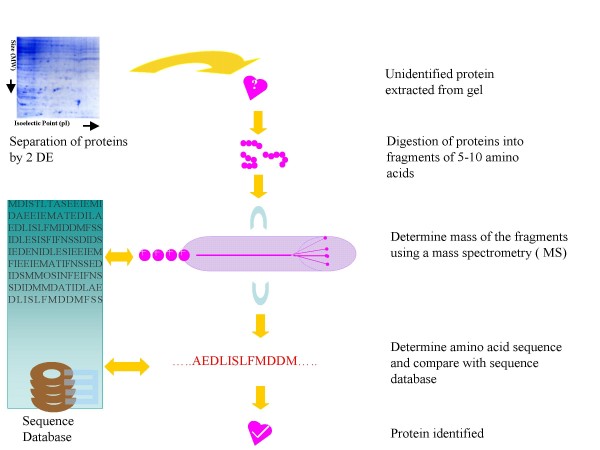
**Schematic representation of protein identification by gel electrophoresis and mass spectrometry (MS)**. The proteins in a sample are separated using a 2 dimensional gel electrophoresis. Each individual protein is extracted from gel and identified by MS.

Two Dimensional (2D) gel electrophoresis is a technique in which proteins in a complex protein mixture (such as cell and tissue samples) are separated according to two dimensions (Figure 1). 2D gel electrophoresis is used primarily to analyze and identify existing proteins in a given sample. 2D gel electrophoresis is a mainstream technology used for proteomic investigations. In this method, proteins are separated in the first dimension according to charge by isoelectric focusing, followed by separation in the second dimension according to molecular weight, using polyacrylamide gel electrophoresis. Then, the gels are stained to visualize separated protein spots using a Coomassie, silver or fluorescent stain. Using this approach, up to several thousand protein spots can be separated and visualized in a single experiment. Gels of different samples are compared and analyzed using computer software. Then, differentially expressed protein spots are excised, digested into fragments and identified using MS.

Recently, a modified 2D protein electrophoresis technique, Differential in Gel Electrophoresis (DIGE), was developed to monitor differences in the proteomic profile of two separate samples. In this method, 2-3 paired samples can be run on the same 2D gel after labeling with each cyanine dyes (Cy2, Cy3 and Cy5). After completing the 2D electrophoresis, the proteins in each sample are detected by a phosphorimager with different fluorescent channels. The different fluorescent images of the same gel are superimposed to identify and quantify differentially expressed proteins. This approach reduces experimental variation, increases quantification accuracy and improves the sensitivity of the technique. The differentially expressed protein spots are then excised from the gel, digested to fragments and identified by MS.

Peptide fragments derived from 2D gel electrophoresis (as well as liquid chromatography) is a precursor to mass spectrometry (MS), a process that identifies the ratio of elemental and isotopic components in a given sample (Figure 1). The principle of MS is that the molecules or proteins in the sample are ionized from the solid to gaseous phase via an ion source. The ions are then separated from each other based on their mass-to-charge (m/z) ratios in the mass analyzer. Finally, the ions are detected, the abundance of each ion is calculated and the structure of the protein is determined by comparing the database against a known protein sequence database [14]. In the past decade, MS has significantly advanced, including improved ionization (matrix assisted laser desorption/ionization (MALDI), electrospray ionization (ESI), surface-enhanced laser desorption ionization (SELDI)) and more sensitive mass analyzers (time-of-flight (TOF), ion trap, and quadrupole). The combination of these systems offers a higher sensitivity (femtomole or picogram), resolution and mass accuracy. In the future, high through-put identification of proteins, broad dynamic range of quantification (10^4 ^to 10^5^) and characterization of post-translational protein modifications will be possible.

#### Detection of known proteins with protein microarray

Protein microarray technology is a powerful emerging analytical strategy for interrogating the proteomes of tissues and cells. As a high-throughput screening platform, protein microarray permits rapid quantitative identification of cancer biomarkers associated with oncogenesis and disease progression. Protein microarray can accelerate the current understanding of cellular differentiation, transformation, angiogenesis, tumorigenesis, and metastasis. This new technology has the ability to 1) quickly elucidate alterations in protein expression levels, 2) detect post-translational modification and mRNA processing events, and 3) dissect molecular networks associated with drug administration or exposure to environmental factors (for example, toxins, infectious agents, or radiation). With the advent of protein microarrays, global profiling cancer signaling network diagnosis and prognosis as well as personalized therapy becomes possible. There are several different types of protein arrays, including reverse phase protein array, antibody array, etc [15] that may be performed on several supporting platforms, including glass slides, membranes, and beads.

**Figure 2:**
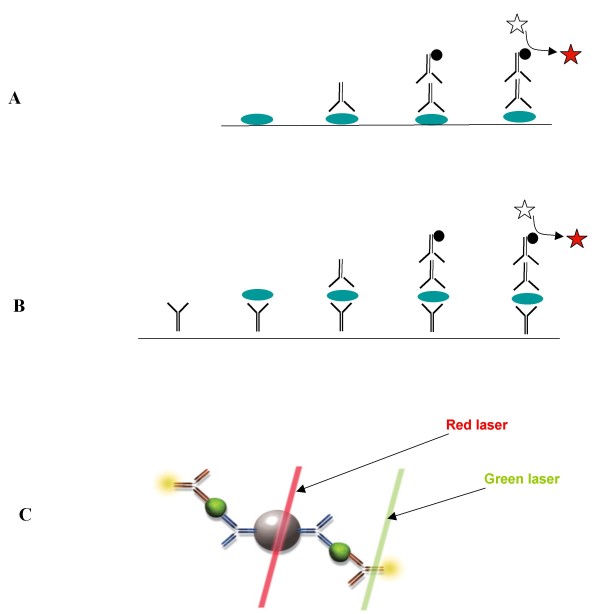
**Antibody-based detection of sample proteins: A) Protein arrays or reverse phase protein arrays**. Proteins are spotted on a support (i.e. glass slide). The primary antibody binds specifically to its protein. The secondary antibody conjugated with HRP (or fluorescence) then bind to the primary antibodies. The substrate is cleaved by HRP to develop detectable color. B) Antibody arrays. Antibodies are spotted on a glass slide and the proteins in the sample are captured on to the glass slide. Another antibody which binds to a different epitope of the protein is used to detect the protein. C) Bead-based array (Luminex platform). The Luminex bead is coated with the capture antibody which binds to the protein in the sample. Another antibody which binds to a different epitope is labeled with fluorescence for the detection. Red laser detects bead and green laser detects the antibody.

**Protein arrays **are typically high-density arrays (>1,000 elements/array) used to identify novel proteins or protein/protein interactions (Figure 2A). The protein library arrayed on the slide can be derived from many possible sources including expression libraries and may contain known as well as unknown elements. Protein arrays can be used to analyze patient samples, including serum and bodily fluids. To detect proteins that are bound to the array, the antibodies must be labeled directly with a fluorophore or a hapten. Alternatively, in some applications, antibodies can be used to detect binding events [16,17].

**Reverse phase protein arrays **are used to profile dozens or hundreds of samples (research or clinical) for the presence of a small number of antigens (Figure 2A). Cell lysates, material from laser capture microdissection, or serum samples are arrayed. This creates an array of "unknowns" that can be probed with a small number of antibodies. Visualization can be performed with a detection antibody linked to a fluorophore or color detection reagent [18].

**Antibody arrays and Microspot ELISA **are used for quantitative profiling of protein expression in cells and clinical samples (Figure 2B). Typically these arrays are low-density (9-100 elements/array). However, the density of the antibody array is expected to increase and will continue to expand due to the availability of a large number of high affinity antibodies. In these arrays, known antibodies are arrayed and used to capture antigens from unknown samples. To detect an antigen that is bound to the array, the antigen is labeled directly with a fluorophore or a second binder/antibody [19]. The latter option creates a sandwich assay similar to a traditional ELISA, but in a microspot format. Thus, the term "microspot ELISA" is used.

**Bead-based array **is a potentially powerful complement to planar arrays. The Luminex bead array system is increasingly used in protein profiling applications (Figure 2C) [20]. The system uses multiple, different fluorescent beads that are spectrally distinguishable and coated with a different capture antibody. The beads are incubated with a sample to allow protein binding to the capture antibodies. The mixture is incubated with a mixture of detection antibodies, each corresponding to one of the capture antibodies. The detection antibodies are tagged to allow fluorescent detection. The beads are passed through a flow cytometer and each bead is probed by two lasers: one to determine the identity of the bead based on the bead's color and another to read the amount of detection antibody on the bead.

**Pathway Array **is an innovative, powerful tool to analyze the expressed proteins with excellent sensitivity and specificity. This is an immunoblot-based assay and was recently developed and validated in the authors' laboratory [21]. It allows for global screening of changes in protein expression and post-translational modification (i.e. phosphorylation). The focus of the Pathway Array is to determine the signaling network that controls cancer development (initiation, promotion, progression and metastasis). The proteins selected for study in the array are highly expressed in cancer cells and are functionally linked to angiogenesis, apoptosis, cell cycle regulation, DNA repair, migration, proliferation, signaling, stem cell association and transcription activity (see Additional file 3).

**Figure 3:**
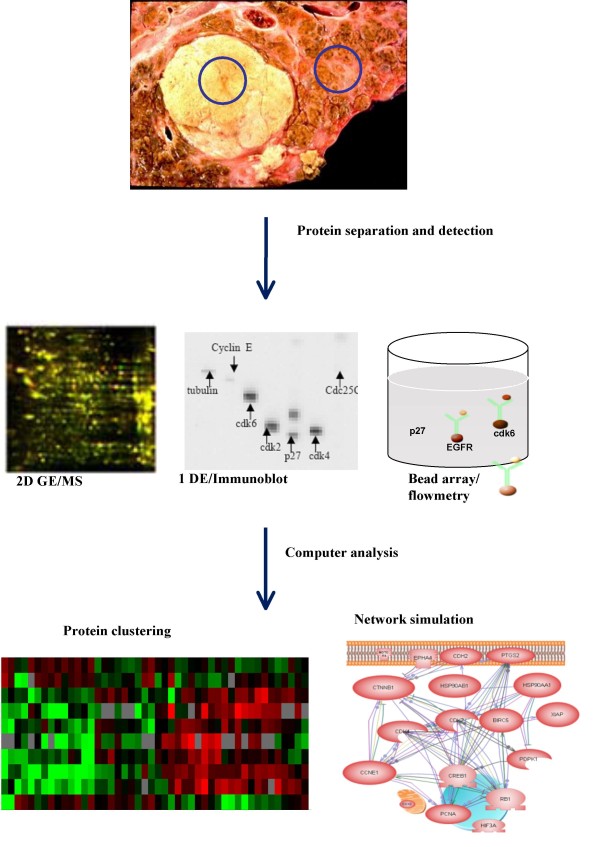
**Flow chart of Pathway Array analysis**. The proteins from tumor and surrounding normal tissues are extracted and separated by 2 dimensional gel electrophoresis (2D GE), 1 dimensional gel electrophoresis (1 DE) or Luminex beads. The presence of specific proteins is detected by mass spectrometry (MS), immunoblot or flow cytometry. Various computer programs are used for data analysis, clustering and network simulation.

The Pathway Array system consists of three integrated components (Figure 3): 1) One or two dimensional gel electrophoresis/multiplex protein immunoblot or bead array; 2) Image acquisition and data analysis; and 3) Computational analysis to integrate the results with known protein-protein, cell signaling and gene regulation cancer biology pathways. Our system measures relative protein levels among different cell lines and tissues. More importantly, the Pathway Array system can assist in identifying global functional changes in the complex signaling network that drives cellular behavior.

**Figure 4:**
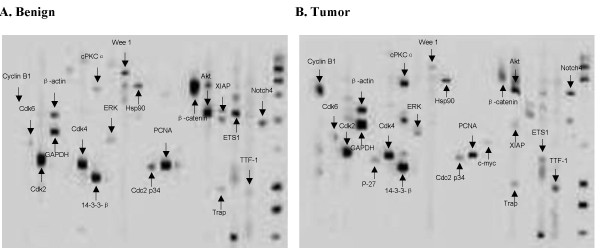
**Representative immunoblots of benign (A) and malignant (B) tissues**. The proteins from benign and malignant tissues were extracted and separated by SDS-PAGE. The proteins were then transferred to a nitrocellular membrane and placed in a manifold which separate the membrane into 20 channels. Each channel was blotted with 2-4 primary antibodies. The secondary antibody was detected by chemiluminescence. The positive signal as well as correct location ensures the correct identification of the proteins.

The Pathway Array (1D gel/immunoblot) can assay several thousands of proteins and phosphoproteins in each sample, depending on the availability of high affinity antibodies (see Additional file 3). Total proteins are extracted from each fresh frozen tissue sample or cell lines and separated using SDS-PAGE. The proteins are then transferred to a nitrocellulose membrane and blotted using a Western blotting manifold that isolates 20 channels across the membrane. Each channel includes 4 antibodies (a total of 80 antibodies) for immunoblot and the proteins specific to the antibody can be detected using a chemiluminescent method (Figure 4). The images can be acquired using the ChemiDoc XRS System (Bio-Rad) and the correct band for each protein/phosphoprotein can be determined by molecular weight. The volume of each band can be recorded. The bound antibodies on the membrane can then be stripped off and blotted with another set of antibodies. This process can be repeated several times so that up to 300-400 antibodies can be blotted using the same membrane.

Pathway Array assay has several important features. The **coverage **of signaling network-related proteins is high with at least one representative protein (typically 2-3) included for each pathway (see Additional file 3). The selection of antibodies was based on previously described functionality in the literature, the Human Protein Atlas http://www.proteinatlas.org/ and commercial availability. It is estimated that approximately 500 genes code for kinases, 1,500 genes code for transcription factors, 400 genes code for G-protein-coupled receptors and 1,200 genes code for candidate cancer biomarkers [22]. Currently, approximately 6,000 antibodies are commercially available, which accounts for nearly 25% of all 21,528 predicted human genes [23].

**Figure 5:**
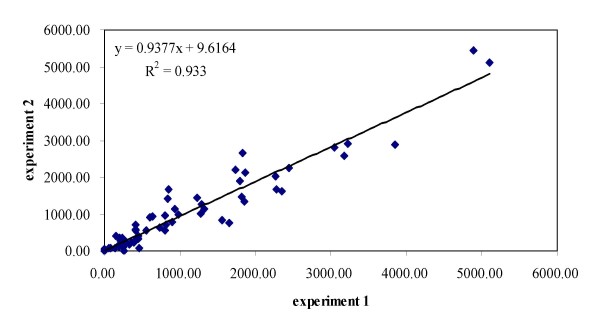
**Correlation between two experiments for detection of 98 protein/phosphoproteins in a breast cancer cell line**. The results showed there is a good correlation between two experiments with R^2 ^= 0.933.

We have validated the sensitivity and specificity of nearly 300 antibodies using different cell lines and human tissues by Pathway Array with an average success rate of 50-70%. The **sensitivity **(or limit of detection) of the assay is about 1 ng for each band by a chemiluminescent detection method (more sensitive compared to conventional Western Blot). The sensitivity can be further improved to 0.1 ng with a fluorescent label (Cy3 and Cy5) and phosphorimager (Typhoon Trio Imager, GE Healthcare). The **specificity **and **accuracy **of the Pathway Array in identifying the correct proteins and phosphoproteins is better than the conventional protein arrays and reverse phase arrays since the correct identification of the protein is based on its molecular weight with a reference to size markers (Figure 4) (Note: false signal for the protein array can be as high as 60%). Over 80-90% of the proteins identified by pathway array can be confirmed by conventional Western blot. The **reproducibility **is also improved with the inter- and intra-run variations: CV = 25% and 35%, respectively, and a R^2 ^= 0.933 between runs (Figure 5). The average **dynamic range **of the assay (using chemiluminescence) is between 10 and 10^4 ^and is very sensitive in detecting differences in protein expression (~2 fold change between two samples). The assay is resistant to **interference **from high abundance proteins (i.e. structural and metabolic proteins which are 10,000~100,000 fold higher than signal transduction proteins) due to the specificity of the antibody and the efficient gel separation. Because of the above features, a higher **discovery rate **of differentially expressed proteins and phosphoproteins (20-40% of the proteins tested) was observed as compared to other gene expression and proteomic-based approaches (2-6% of the mRNA expression array or 2D/MS). For example, we studied 39 pairs of non-small cell lung cancer and surrounding normal tissues to identify differentially expressed proteins using Pathway Array. Among 108 proteins and phosphoproteins tested, 59 were detected and 21 were differentially expressed with p < 0.05 as determined by SAM analysis. The detection rate was 55% and the rate of discovering differentially expressed proteins was 20%. The higher detection and discovery rates of the Pathway Array are due to the inclusion of antibodies that are highly relevant in carcinogenesis.

### Computational Methods For Proteomic Array Data Analysis

Currently, a bioinformatic method specifically designed for high density protein expression array analysis is unavailable. However, some statistical tools developed for genomic microarray can be used for protein arrays. Examples of statistical tools that can be used for protein arrays are listed below.

**BRB-Array Tools **is an integrated software package for the analysis of genomic microarray data http://linus.nci.nih.gov/BRB-ArrayTools.html. BRB-Array Tools is an add-on to Excel and provides an user friendly platform to perform ANOVA functions, cluster genes and samples, functional and predictive sample classifications, and data visualization tools [24]. The package is very portable and has unrestricted use with any particular array platform, scanner, image analysis software or database. BRB-Array Tools identifies differentially expressed genes across groups (also referred to as "Class Comparison") using reliable statistical methods designed to better manage the false discovery rate (FDR). It also constructs and evaluates multivariate predictors for classifying unknown samples into groups based on gene expression profiles (also referred to as "Class Prediction"). For protein expression array analysis, the data set of protein expression signal intensities can be imported to BRB-ArrayTools in an Excel format. The computations are performed by sophisticated statistical tools external to Excel, including ANOVA for identification of genes differentially expressed amongst the groups (two or more groups) and t-test or F-test (paired groups). The outputs are gene rank lists based on statistical tests and figures based on visualization tools, including heat map and Multi-Dimensional Scaling which reduces high dimensional data to graphical displays. We have successfully applied BRB-Array Tools for our Pathway Array data analysis (see next section).

**Figure 6:**
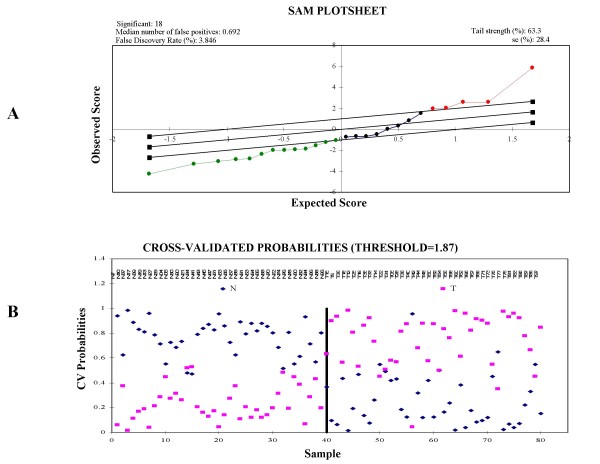
**Statistical analysis of Pathway Array results**. A. SAM output of breast cancer data which showed a FDR 3.85% in 18 differently expressed proteins between tumor and benign tissues. B. PAM output of Cross-validated probabilities of 9 differently expressed biomarkers between lung cancer (T) and normal tissues (N).

**Significance Analysis of Microarrays **(SAM) is a supervised learning software for genomic array data mining http://www-stat.stanford.edu/~tibs/SAM/. SAM is a statistical tool for identifying significant genes in a set of expression microarray experiments. It can also be applied to data from Oligo or cDNA arrays, SNP arrays, protein arrays, etc (Figure 6A) [25]. SAM correlates expression data to clinical parameters including treatment, diagnosis categories, survival time, paired (before and after), quantitative (e.g. tumor volume) and one-class. Both parametric and non-parametric tests can be performed by SAM. SAM can perform the automatic imputation of missing data via nearest neighbor algorithm. The adjustable threshold determines the number of genes called significant. SAM uses data permutations to provide an estimate of FDR for multiple testing. The output of gene lists in Excel workbook form can easily be exported into TreeView, Cluster or other software. Finally, the genes are web-linked to the Stanford SOURCE database.

**Prediction Analysis of Microarrays **(PAM) is a statistical technique for class prediction and survival analysis from gene expression data using nearest shrunken centroids http://www-stat.stanford.edu/~tibs/PAM/ (Figure 6B). The method of nearest shrunken centroids identifies subsets of genes that best characterizes each class [26]. The technique is general and can be used in many other classification problems. For survival outcomes, PAM uses prediction by the 'supervised principal components' method. PAM incorporates reliable statistical methods designed to better manage the FDR. PAM estimates prediction error via cross-validation and provides a list of significant genes whose expression characterizes each diagnostic class. The data from cDNA and oligo microarrays, protein expression data and SNP chip data can used for PAM analysis.

#### Signaling network construction

Although computational modeling platforms will soon become standard tools in constructing signaling networks for clinical applications, comprehensive tools are still not presently available. To establish 2-dimensional or even 3 -dimensional signal signaling networks in cancer cells requires the integration of mathematical, computational, biology and clinical sciences. The data available from genomic, proteomic and biochemical experiments creates a framework for signaling network construction. Furthermore, the integration of data from of other genomic studies included mRNA expression, SNP, CNV, methylation etc. is necessary to establish a comprehensive signaling network.

**Figure 7:**
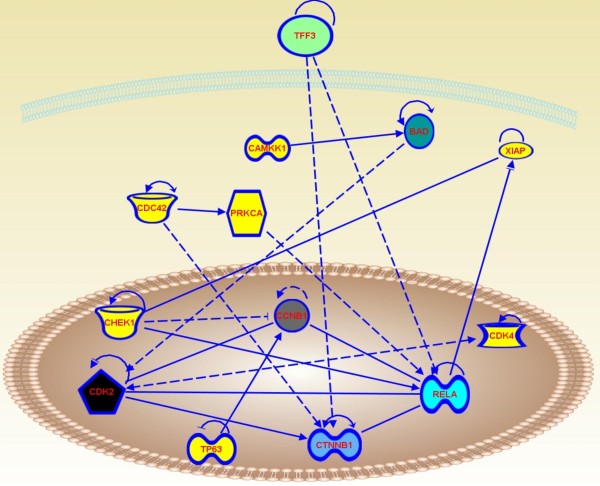
**Schematic representation of the signaling network of differentially expressed proteins**. The figure was created using Ingeniuty and shows the connectivity between nodes (i.e. TCP1 and CDC42) and hubs (i.e. CCNB1, CTNNB1 and RELA). Solid lines indicate direct interaction. Dashed lines indicate indirect interaction. Arrows indicate stimulation. Bars indicate inhibition.

The network construction is conceptually straightforward: nodes represent proteins or genes and hubs represent the central regulators that control other nodes through the links which connects between node-node, node-hub, and hub-hub (Figure 7). The most common mathematic modeling tool is **Bayesian network analysis **[27]. A Bayesian network is a probabilistic model that consists of two parts: a dependency structure and local probability models. The dependency structure specifies how the variables are related to each other by drawing directed edges between the variables without creating directed cycles. Each variable depends on a possibly empty set of other variables, termed the "parents." The local probability model specifies how the variables depend on the parents.

Several computer programs are available for simple prediction of signaling network and graphic presentation. **Weighted Co-expression Analysis **is used to explore molecular interaction networks across RNA expression in different samples in microarray datasets [28]. In this model, the network construction is based on the concept that nodes represent genes and nodes are connected if the corresponding genes are significantly co-expressed across appropriately chosen tissue samples. The co-expression networks can be organized into modules of system level functionality for coordinated gene expression. **Category analysis **and **Gene set enrichment analysis (GSEA) **provide pathway enrichment tools to help interpret datasets [29]. This approach designed to detect the categories, or sets, of genes where there are potentially small but coordinated changes in the expression of groups of functionally related genes. **Ingenuity Pathway Analysis**, a commercial software, provides a link to database derived from literature to find function and pathways for microarray analysis http://www.ingenuity.com/products/pathways_analysis.html. Ingenuity Pathway Analysis is an web-based application that enables users to analyze, integrate, and understand data derived from gene expression, microRNA, SNP and proteomic microarray [30]. The capabilities of Ingenuity Pathway Analysis are to: 1) rank the genes and proteins in a dataset according to the characteristics that make a gene product a biologically plausible candidate biomarker; 2) measure whether a particular gene or protein is detectable in sentinel tissues (e.g., blood, bone marrow), urine and other bodily fluids; 3) select parameters that are most relevant to a biomarker discovery project; 4) elucidate mechanisms linking potential markers to the disease or biological process of interest; and 5) generate a list of candidate markers unique to one treatment or disease, or common across all treatments.

Some existing signaling network databases are also available including KEGG pathway http://www.genome.jp/kegg/pathway.html and BioCarta pathways http://www.biocarta.com/genes/allpathways.asp. These networks were constructed based on published literature and databases, such as Entrez Gene http://www.ncbi.nlm.nih.gov/entrez/query.fcgi?db_gene and Gene Ontology, which provide molecular function, biological process and cellular location. Gene Ontology is a major bioinformatics initiative that aims to standardize the representation of gene and gene product attributes across species and databases http://www.geneontology.org/GO.tools.shtml. The project provides a controlled vocabulary of terms for describing gene product characteristics and gene product annotation data from GO Consortium members, as well as tools to access and process this data.

### Research and Clinical Applications for Pathway Array

The Pathway Array has broad applications in translational research and clinical utilities, including discovering diagnostic and prognostic biomarkers, identifying novel therapeutic targets, and providing tools for future personalized therapy. The following are examples of Pathway Array applications in different cancers.

#### Discovery of diagnostic biomarkers

**Figure 8:**
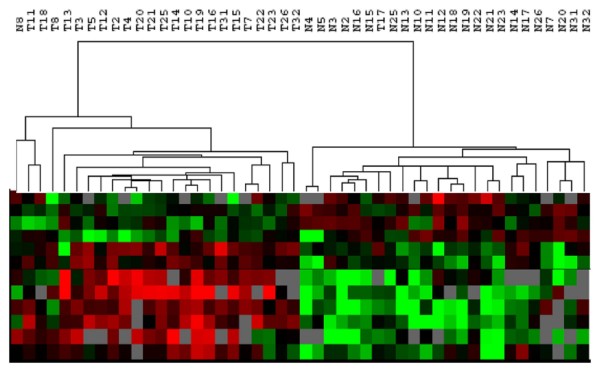
**Classifying benign (N) and malignant (T) breast tissues using 12 signaling-related proteins**. Based on the expression pattern, the tumors (left) were separated from the benign tissues (right) with only 1 benign (N8) and one tumor (T17) misclassified. Red: increased expression. Green: decreased expression. Black: no change. Gray: no expression.

Breast cancer is the second leading cancer death in women. Breast cancer research in the past decade has advanced our understanding of breast cancer biology and improved diagnosis and treatment. Most advancement has occurred from studies of breast cancer using techniques such as, cytogenetics, gene expression array, SNP array, copy number variation, DNA methylation, etc. We recently completed a comparative study of 39 breast cancer patients that examined the differences in expression pattern of invasive ductal carcinoma and the surrounding normal tissues. The Pathway Array data was analyzed using BRB-Array Tools and SAM as described above. Of 160 proteins/phospho-proteins tested (see Additional file 3, for a partial list of the antibodies), 56 are differentially expressed with statistical significance (p < 0.05), including but not limited to: Twist, Fas, PCNA, PTEN and cyclin B1. Some proteins are only overexpressed in tumors (i.e. PTEN), while others are down-regulated in tumor tissues (i.e. cyclin B1). We further analyzed the expression data using PAM and identified 12 proteins that best characterize tumor and normal class of breast tissues. Using these proteins, the tumor and normal tissue was distinguished with 96% accuracy in 24 pairs of breast cancer and normal tissue specimens (Figure 8).

**Figure 9:**
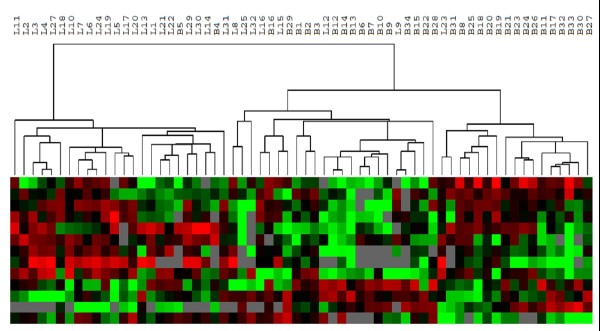
**Classify lung cancer (L) and breast cancer (B) using 13 signaling proteins (30 lung cancer samples and 34 breast cancer samples)**. Based on the expression pattern, the lung cancers (left) were separated from the breast cancers (right) with 3 breast cancers and 3 lung cancers misclassified. Red: increased expression. Green: decreased expression. Black: no change. Gray: no expression.

We further tested to see if selected signaling proteins can separate breast cancer from other cancers, such as lung cancer, since histologically distinguishing breast cancer from other types of cancers may be a diagnostic challenge in certain circumstances. Our results showed that using 13 differentially expressed proteins between breast cancer and lung cancer, we were able to separate breast cancer from lung cancer with 91% accuracy (Figure 9). These results suggested that breast cancer and lung cancer have distinct dysregulation and activation patterns, probably due to the different mechanisms of carcinogenesis.

#### Discovery of prognostic biomarkers

Most studies that predict non-small cell lung cancer survival used a genomic microarray platform. For example, Chen *et al *showed that 16 genes from an initial microarray study and risk score analysis correlated with survival among patients with NSLC [31]. The authors subsequently selected five genes (DUSP6, MMD, STAT1, ERBB3, and LCK) for RT-PCR and decision-tree analysis. The five-gene signature was an independent predictor of relapse-free and overall survival. Fan et al. showed that a 13 gene profile associated with the vascular endothelial growth factor (VEGF) subnetwork, including VEGF, ANGPTL4, ADM and the monocarboxylic acid transporter SLC16A3, can predict distant metastasis and poor outcomes [32]. These results suggest that activation of certain signaling network may correlate with a poor prognosis.

**Figure 10:**
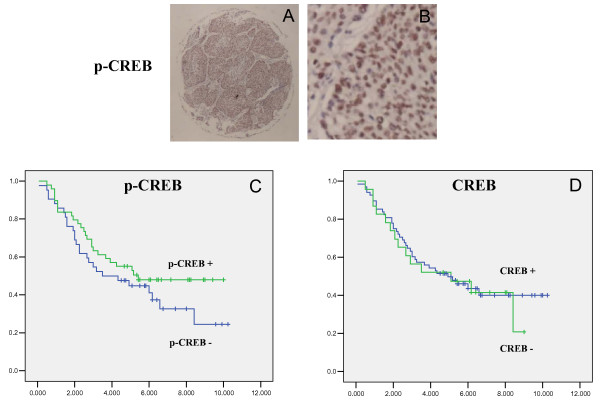
**Prognostic value of phosphorylated CREB (p-CREB) for lung cancer**. A and B. Immunochemistry staining of p-CREB on a lung cancer tissue microarray (A: 10× magnification; B: 400× magnification). Positive stains (brown) of p-CREB were seen in tumor cell nuclei. C and D. Survival analysis using p-CREB (C) and total CREB (D) expression. The results showed that p-CREB expression correlated better survival. In contrary, expression of unphosphorylated CREB had not prognostic value.

We recently analyzed the expression of p-CREB in lung cancer using Pathway Array technique and found that it differentially expressed in 56.4% of NSCLC compared with surrounding normal tissue in our cohort of 39 patients. We further tested the expression of p-CREB in a NSCLC tissue microarray (n = 91) using immunohistochemical staining method and the expression pattern was correlated with survival (Figure 10A and 10B). Our results showed that p-CREB was expressed in the nucleus in 63% of NSCLC and the increased expression of p-CREB correlated with a good prognosis (Figure 10C and 10D).

#### Discovery of potential therapeutic targets

Hepatocellular carcinoma (HCC) is the fifth most common malignancy and the third leading cause of cancer death in the world, with the five-year survival rate approaching 7% [33]. Treatments of HCC include surgical resection and transplantation, ablation and transarterial chemoembolization, and systemic chemotherapy [34,35]. However, except for surgical resection/transplantation of early stage HCC, the survival time is not significantly prolonged by any of these treatments. Therefore, development of newer therapeutic targets for HCC treatment is urgently needed.

We studied 10 tumor tissues and paired non-tumor tissues from 10 hepatitis-related HCC patients. Among the 44 antibodies tested, 23 proteins and phosphoproteins were detected and 22 had a more than 2-fold change between the cancerous and normal tissues. Of these proteins, XIAP and CDK6 were highly expressed in tumors as compared with surrounding tissues (54% and 46% tumors, respectively). XIAP is a member of the IAP family (inhibitor of apoptosis), which inhibits a subset of caspases (i.e. caspase 3 and 9). CDK6 activates cyclin D1 and the cyclin D1/CDK6 complex activates pRB and E2F which controls the cell cycle progression from the mid-G1 to S phase. Therefore, increased expression of XIAP and CDK6 in HCC may result in decreased apoptosis and increased cell proliferation.

In order to determine if XIAP and CDK6 can be therapeutic targets, we applied siRNA technology to silence the expression of XIAP and CDK6 in HCC cells. Our results showed a significant reduction in cell viability by both XIAP and CDK6 specific siRNAs and the cause of cell death was necrosis (i.e. PI positive cells) rather than apoptosis (Annexin positive cells) (see Additional file 4). These results indicate that both XIAP and CDK6 are important for HCC cell survival. We further tested that small molecules specific for these targets, including embolin for XIAP and flavopiridol for CDKs. Both molecules showed a significant inhibition of HCC cell growth, suggesting that both XIAP and CDK6 can be potential targets for HCC treatment.

#### Detection of signaling activities for personalized therapy

Currently, the treatment of most cancers is based on the tissue types and clinical stages. This approach is often ineffective due to the heterogeneity of the tumors. Recently, the use the signaling network approach to break down complex oncogenic signaling networks into basic units, or modules, of signaling activity (e.g., a protein phosphorylating another protein to activate its kinase activity) and demonstrate that gene expression signatures based on these modules can predict the effectiveness of pathway-specific therapeutics [36].

As stated above that current systemic chemotherapy for HCC is ineffective [34,35]. A recent study showed that targeted therapy with molecules, such as sorafenib which inhibits multiple tyrosine kinase receptors (RAS/VEGFR) [37], may offer some benefit with this deadly disease (~3 months improvement of survival). A reason for the limited benefit of signal pathway based treatment is the redundancy and compensation of the signaling network in HCC. Our recent study showed inhibition of XIAP and CDK6 reduced HCC cell proliferation. However, a significant reorganization of the signaling network observed, including down regulation of tumor suppressors (p-p53 and CHK1 when XIAP silenced or p-RB when CDK6 silenced) and upregulation of tumor promoting proteins (ETS1 when XIAP silenced or p-CREB when CDK6 silenced), which may confer the growth benefit for cancer cells. Therefore, it is conceivable that inhibition of a main pathway and an associated compensatory pathways, the efficacy of chemotherapy will be significantly improved.

Another potential cause of treatment failure is the phenotypical heterogeneity of HCC that results from heterogeneous activation of cancer signaling network [38]. Our study showed a significant variation in signaling transduction protein expression in different patients (see Additional file 5). For example, CDK6 was only expressed in patient A while ERK1/2 was expressed in patient B and C. In this case, if flavopiridal (a pan-CDK inhibitor) is used to treat these patients, the drug may not have been effective in patients B, C and D. On the other hand, when sorafenib (a RAS/ERK pathway inhibitor) is used, the drug may not have been effective for patient A and D. Therefore, assessing the signaling pathway/network before beginning treatment to identify patients that may benefit from targeted therapies may improve the response rate.

## Conclusion

Cancer is a complex disease that results from dysregulation of signaling networks caused by the genetic and epigenetic alterations in cells. Therefore, determining the underlying signaling network changes in cancer not only help to understand the molecular mechanism of carcinogenesis but also to identify the signature of signaling networks characteristic for specific cancer types that can be used for diagnosis, prognosis and guidance for targeted therapy. The scientific community will see a significant advancement in cancer signaling network field in the next 5-10 years. Pathologists and laboratory scientists are in the unique position to translate their knowledge of cancer signaling network biology into relevant clinical practice. Conceivably, "-omics" tools, such as DNA and protein microarray, can be successfully used as tissue-based diagnostic and prognostic tools in the future. One ideal model is that cancer patients may visit the oncologist, cytopathologist or radiologist to perform a fine needle aspiration biopsy (FNA) of the tumor. Then, the FNA materials are examined by a cytopathologist for tumor cells and analyzed by a molecular pathologist to reconstruct the signaling network using "-omics tools." This information would be integrated so that the oncologist can use the signaling network information, in addition to clinical and pathological data, to determine the prognosis and customize the treatment (i.e. personalized therapy).

## List of abbreviations

SNP: single nucleotide polymorphism; CNV: copy number variations (CNV); 2D: Two dimensional; LC: liquid chromatograph; MS: mass spectrometry; DIGE: Differential in Gel Electrophoresis (DIGE); MALDI: matrix assisted laser desorption/ionization; ESI: electrospray ionization; SELDI: surface-enhanced laser desorption ionization; TOF: time-of-flight; SAM: Analysis of Microarrays; HCC: Hepatocellular carcinoma.

## Conflict of interests

The authors declare that they have no competing interests.

## Authors' contributions

DYZ conceived the study and participated in study design and manuscript preparation. FY supervised and coordinated the study. LG participated in breast cancer study. XLL performed statistical analysis. XZ performed array analysis and statistical analysis. YC participated in HCC study. HW participated in breast cancer study. LW participated in lung cancer study. JW participate in study coordination and manuscript preparation. DS participate in breast cancer study. WL participate in lung cancer study. HX participated in breast cancer study. BJ participated in HCC study. WJZ performed statistical analysis. JHW performed pathway analysis. PL participated in study design and manuscript preparation. All authors read and approved the final manuscript.

## Supplementary Material

Additional file 1 **Microarray technologies used in genomic and epigenetic analysis**. Important features of genomic and epigenetic arrays. Click here for file

Additional file 2 **Comparison of different proteomics-based techniques**. Advantage and disadvantages of various proteomic technologies. Click here for file

Additional file 3 **List of antibodies included in the immunoblot array (partial list)**. Relvant signaling proteins used in Pathway Array analysis. Click here for file

Additional file 4 **Effect of Cdk6 and XIAP silencing on cell viability, cell cycle distribution and necrosis**. Example of functional relevance of signaling proteins. Click here for file

Additional file 5 **The expression of signaling transduction proteins in HCCs from 4 patients**. Examples of the expression level of signaling proteins in HCC. Click here for file
