# Supplementary material for: Proteomics, pathway array and signaling network-based medicine in cancer
Source: Cell Div. 2009 Oct 28;4:20. doi: 10.1186/1747-1028-4-20 (PMC2780394; doi:10.1186/1747-1028-4-20)
Supplement: Additional file 1 — Microarray technologies used in genomic and epigenetic analysis. Important features of genomic and epigenetic arrays. [file 1747-1028-4-20-S1.doc]

**Additional file 1, Microarray technologies used in genomic and epigenetic analysis:**

| **MICROARRAY** | **CHARACTERISTIC FEATURE** |
| --- | --- |
| **GENE EXPRESSION ARRAY [5]** | - Simultaneous monitoring of  expression levels for >45,000 transcripts to study the effects of certain treatments, diseases,  and developmental stages on gene expression using high-density arrays - Does not detect splicing variants as probes are designed to interrogate the 3' end of the transcripts - Requires as little as 2 ug of starting mRNA for reverse transcription and labeling |
| [**ALTERNATIVE SPLICING**](http://en.wikipedia.org/wiki/Alternative_splicing)**ARRAY [6]** | - Used to assess the expression of alternative splice forms of thousands of genes - Exon arrays have a different design that employs probes designed to detect each individual exon for known/predicted genes - Used to detect different splicing isoforms - Requires as little as 100 ng of starting mRNA |
| **MICRORNA ARRAY [7]** | - A high-throughput technique to assess cancer-specific expression levels for hundreds of miRNAs in a large sample numbers - 500 human miRNAs have been recorded in mirbase (<http://microrna.sanger.ac.uk/sequences/>) - miRNAs are involved in gene expression regulation. |
| [**SNP**](http://en.wikipedia.org/wiki/SNP_array) **ARRAY [8]** | - - Used to identify single nucleotide polymorphisms among alleles within or between populations   - Evaluates germline mutations in individuals or somatic mutations in cancers, assessing loss of heterozygosity, or genetic linkage analysis   - Can measure more than 900,000 SNPs in the whole genome |
| [**COMPARATIVE GENOMIC HYBRIDIZATION**](http://en.wikipedia.org/wiki/Comparative_genomic_hybridization) **ARRAY[9]** | - - - Used to detect loss, gain and amplification of copy number at the chromosomal level     - Can detect small gains and losses, e.g.  Inter-marker distance of ~100-700 base pairs     - Combined with SNP array, can be used for genome wide association studies |
| **CHIP-ON-CHIP ARRAY [10]** | - - - Combines chromatin immunoprecipitation (chip) with microarray technology ("chip").     - High throughput (genome-wide) identification and analysis of DNA fragments bound by specific proteins such as histones, transcriptional factors.     - Used to investigate interaction between protein and DNA     - Identifies binding sites of DNA-binding proteins in a genome-wide basis. |
| **DNA METHYLATION ARRAY [11]** | - - DNA methylation is an abnormal heritable epigenetic modification process occurring in cancer cells whereby cpg dinucleotides are methylated at the C5 position of cytosine   - The methylation of the 5’ regulatory regions of genes results in gene silencing   - Methylated DNA are captured with 5-methlycytidine antibody or methyl binding domain proteins and hybridized to a DNA array which contains 385k to 2.1M probes |
