# Supplementary material for: Proteomics, pathway array and signaling network-based medicine in cancer
Source: Cell Div. 2009 Oct 28;4:20. doi: 10.1186/1747-1028-4-20 (PMC2780394; doi:10.1186/1747-1028-4-20)
Supplement: Additional file 2 — Comparison of different proteomics-based techniques. Advantage and disadvantages of various proteomic technologies. [file 1747-1028-4-20-S2.doc]

**Additional File 2, Comparison of different proteomics-based techniques**

| **METHOD DESCRIPTION** | **ADVANTAGES** | **DISADVANTAGES** | **SENSITIVITY** |
| --- | --- | --- | --- |
| **2D GEL ELECTROPHORESIS/MASS SPECTROMETRY** | | | |
| - Separation of complex proteins via 2D gel electrophoresis based charge and size - Major protein identification by MS - Detects about 2000-2500 spots/gel | - Ability to identify unknown proteins - Detects protein modification (phosphorylation and methylation) - Used for various biological samples, including tissue, blood and other biological fluids | - Proteins expressed at low abundance may be missed - Unsuited for diagnostic application - Limited reproducibility and high rate of false identification - Limited dynamic  range - semi-quantitative | - Detection sensitivity is in the nanogram range (50 ng/spot for Coomassie Blue; 1 ng/spot for silver stain) - Using fluorescent 2D-differential gel electrophoresis (2D-DIGE), sensitivity improves by 10 fold (CyDye label) |
| **LIQUID CHROMATOGRAPHY/MASS SPECTROMETRY** | | | |
| - LC to separate proteins in a sample, with sequential LC for improved separation efficiency - MS to systematically identify the major proteins - Detects over 1000 proteins/run | - Ability to identify unknown proteins - improved separation efficiency compared to 2D gel - Used for various biological samples, including tissue, blood and other biological fluids | - Proteins expressed at low abundance may be missed - Unsuited for diagnostic application - Limited reproducibility and high rate of false identification - Limited dynamic  range - semi-quantitative | - Detection sensitivity is in the nanogram range or ~20 cells - 1% false positive rate |
| **PROTEIN ARRAY** | | | |
| - Individual protein immobilization on a solid-support (glass or membrane) - Individual proteins identified by labeled antibodies - Detects over 1000 proteins/array | - High sensitivity and specificity - Good quantitation range - High throughput/density amenable for automation - Economical and low sample consumption - Lots of data from single experiment - Software and hardware tools may be shared with DNA microarray | - Limited protein availability from complex protein production process (expression and purification) - Limited access to a large number of affinity antibodies for detection. | - Detection sensitivity is in the ng/ml range |
| **REVERSE PHASE PROTEIN ARRAY** | | | |
| - Multiple whole-cell or tissue lysate immobilization on individual spots on a solid support (similar to tissue microarray format) - Presence of specific proteins are detected by antibody - Detects < 100 proteins/array | - Highly sensitive detection of proteins - High throughput, i.e. a large number of samples on one slide - Minimal sample required - Reduced number of antibodies needed to detect protein | - Detection sensitivity may be compromised from loss native protein conformation when surface spotted - Limited sensitivity to detect low abundance proteins - Specificity may be compromised from non-specific antibody binding (i.e. potential for high background) - Limited number of available signaling protein-specific antibodies | - Detection sensitivity is in the picogram range - Increased sensitivity - Using laser capture microdissection, lysates can be analyzed with as few as 10 cells |
| **ANTIBODY ARRAY** | | | |
| - Capture antibodies are spotted and fixed on a solid surface - Proteins (antigens) are captured on the array surface and detected by a second antibody specific for a different epitopes than capture antibody (sandwich format) - Detects < 100 proteins/array | - Highly specific from dual antibody detection - Highly sensitive - High throughput and amenable for automation - Possible to detect protein modifications (phosphorylation, methylation, etc) by modification-specific antibodies - Suitable for clinical applications | - Protein complexity and denaturation may affect antigen-antibody interaction - Need for high-affinity and specific antibodies  for capture and detection - Limited dynamic  range of 2 or 3 orders of magnitude | - Detection sensitivity is in the low pg/ml range |
| **PATHWAY ARRAY** | | | |
| - Complex proteins in a sample (cells or tissue) are separated via gel electrophoresis - Proteins then transfers to nitrocellular membrane - Proteins detected by multichannel immunoblot (similar to Western Blot) - Detects up to 300 proteins/run | - Highly sensitive with detection of low abundance proteins - Highly specific (as determined by immunoreactivity and size) - High accuracy and reproducibility - Minimal antibody required for each sample - Detects protein modifications (phosphorylation, methylation, etc) | - Limited availability of signaling-related antibodies - Relative low through- put (one sample per gel - Limited dynamic  range of 2 or 3 orders of magnitude | - Detection limit of 1 ng for each protein with chemiluminescence; 0.1 ng with fluorescence - Linear detection range is 100 fold for ECL and 1,000 for fluorescence. |
| **BEAD-BASED ARRAY** | | | |
| - Either capture antibody or proteins are coated on beads - Detection of proteins by labeled antibodies (similar to antibody array or ELISA) - Detects 50-100 proteins/run | - Highly sensitive and specific - High throughput and amenable for automation - Detects protein modifications (phosphorylation, methylation, etc) by modification specific antibodies - Suitable for clinical applications | - Protein complexity and denaturation affecting antigen-antibody interaction - Need for high-affinity and specific antibodies for capture and detection - Limited dynamic range of 2 or 3 orders of magnitude | - Detection limit is sufficient to capture low abundance protein analytes down to the pg/mL range |
| **METHOD DESCRIPTION** | **ADVANTAGES** | **DISADVANTAGES** | **SENSITIVITY** |
| **2D GEL ELECTROPHORESIS/MASS SPECTROMETRY** | | | |
| - Separation of complex proteins via 2D gel electrophoresis based charge and size - Major protein identification by MS - Detects about 2000-2500 spots/gel | - Ability to identify unknown proteins - Detects protein modification (phosphorylation and methylation) - Used for various biological samples, including tissue, blood and other biological fluids | - Proteins expressed at low abundance may be missed - Unsuited for diagnostic application - Limited reproducibility and high rate of false identification - Limited dynamic  range - semi-quantitative | - Detection sensitivity is in the nanogram range (50 ng/spot for Coomassie Blue; 1 ng/spot for silver stain) - Using fluorescent 2D-differential gel electrophoresis (2D-DIGE), sensitivity improves by 10 fold (CyDye label) |
| **LIQUID CHROMATOGRAPHY/MASS SPECTROMETRY** | | | |
| - LC to separate proteins in a sample, with sequential LC for improved separation efficiency - MS to systematically identify the major proteins - Detects over 1000 proteins/run | - Ability to identify unknown proteins - improved separation efficiency compared to 2D gel - Used for various biological samples, including tissue, blood and other biological fluids | - Proteins expressed at low abundance may be missed - Unsuited for diagnostic application - Limited reproducibility and high rate of false identification - Limited dynamic  range - semi-quantitative | - Detection sensitivity is in the nanogram range or ~20 cells - 1% false positive rate |
| **PROTEIN ARRAY** | | | |
| - Individual protein immobilization on a solid-support (glass or membrane) - Individual proteins identified by labeled antibodies - Detects over 1000 proteins/array | - High sensitivity and specificity - Good quantitation range - High throughput/density amenable for automation - Economical and low sample consumption - Lots of data from single experiment - Software and hardware tools may be shared with DNA microarray | - Limited protein availability from complex protein production process (expression and purification) - Limited access to a large number of affinity antibodies for detection. | - Detection sensitivity is in the ng/ml range |
| **REVERSE PHASE PROTEIN ARRAY** | | | |
| - Multiple whole-cell or tissue lysate immobilization on individual spots on a solid support (similar to tissue microarray format) - Presence of specific proteins are detected by antibody - Detects < 100 proteins/array | - Highly sensitive detection of proteins - High throughput, i.e. a large number of samples on one slide - Minimal sample required - Reduced number of antibodies needed to detect protein | - Detection sensitivity may be compromised from loss native protein conformation when surface spotted - Limited sensitivity to detect low abundance proteins - Specificity may be compromised from non-specific antibody binding (i.e. potential for high background) - Limited number of available signaling protein-specific antibodies | - Detection sensitivity is in the picogram range - Increased sensitivity - Using laser capture microdissection, lysates can be analyzed with as few as 10 cells |
| **ANTIBODY ARRAY** | | | |
| - Capture antibodies are spotted and fixed on a solid surface - Proteins (antigens) are captured on the array surface and detected by a second antibody specific for a different epitopes than capture antibody (sandwich format) - Detects < 100 proteins/array | - Highly specific from dual antibody detection - Highly sensitive - High throughput and amenable for automation - Possible to detect protein modifications (phosphorylation, methylation, etc) by modification-specific antibodies - Suitable for clinical applications | - Protein complexity and denaturation may affect antigen-antibody interaction - Need for high-affinity and specific antibodies  for capture and detection - Limited dynamic  ranges of 2 or 3 orders of magnitude | - Detection sensitivity is in the low pg/ml range |
| **PATHWAY ARRAY** | | | |
| - Complex proteins in a sample (cells or tissue) are separated via gel electrophoresis - Proteins then transfers to nitrocellular membrane - Proteins detected by multichannel immunoblot (similar to Western Blot) - Detects up to 300 proteins/run | - Highly sensitive with detection of low abundance proteins - Highly specific (as determined by immunoreactivity and size) - High accuracy and reproducibility - Minimal antibody required for each sample - Detects protein modifications (phosphorylation, methylation, etc) | - Limited availability of signaling-related antibodies - Relative low through- put (one sample per gel - Limited dynamic  ranges of 2 or 3 orders of magnitude | - Detection limit of 1 ng for each protein with chemiluminescence; 0.1 ng with fluorescence - Linear detection range is 100 fold for ECL and 1,000 for fluorescence. |
| **BEAD-BASED ARRAY** | | | |
| - Either capture antibody or proteins are coated on beads - Detection of proteins by labeled antibodies (similar to antibody array or ELISA) - Detects 50-100 proteins/run | - Highly sensitive and specific - High throughput and amenable for automation - Detects protein modifications (phosphorylation, methylation, etc) by modification specific antibodies - Suitable for clinical applications | - Protein complexity and denaturation affecting antigen-antibody interaction - Need for high-affinity and specific antibodies for capture and detection - Limited dynamic ranges of 2 or 3 orders of magnitude | - Detection limit is sufficient to capture low abundance protein analytes down to the pg/mL range |
