# Supplementary material for: Proteomics, pathway array and signaling network-based medicine in cancer
Source: Cell Div. 2009 Oct 28;4:20. doi: 10.1186/1747-1028-4-20 (PMC2780394; doi:10.1186/1747-1028-4-20)
Supplement: Additional file 3 — List of antibodies included in the immunoblot array (partial list). Relvant signaling proteins used in Pathway Array analysis. [file 1747-1028-4-20-S3.doc]

**Additional file 3, List of antibodies included in the immunoblot array (partial list)**

_____________________________________________________________________________

**Cell signaling:** ERK1/2, p-ERK1/2 (Thr202/Tyr204), Akt, p-AKT (Ser473), HGF, HGFR, pHGFR (Y1234/Y1235), IGF, IGFR, TGF, TGFR, Notch 4, Notch 1, p38, p-p38 (Thr180/Tyr182), JNK, p-JNK (Thr183/Tyr185), FGFR, p-FGFR (Tyr653/654), VEGFR, p-VEGFR (Tyr951), PKC, p-PKCalpha (Ser657), p-PKCα/β(Thr638/641), PTEN, p-PTEN (Ser380), PI3K, Ras, Raf, EGFR, p-EGFR (Tyr1068), p-EGFR (Tyr1148), p-EGFR (Tyr1173), Her2, p-Her2 (Tyr1221/1222), PDK1, p-PDK1 (Ser241), mTor, p-mTor (Ser2448), HSP90, NF-kB, IKB, c-Kit, c-Kit (Tyr719), PDGFR, GSK3, beta-catenin, p-be,ta-catenin (Ser33/37/Thr41), stat3, p-stat3 (Ser727), stat5, p-stat5 (Tyr694), smad, p-smad (Ser463/465), CREB, p-CREB (Ser133), Frizzled receptor, APC

**Cell Growth/Cell Proliferation:** Rb, P21, P27, P15, P16, P18, P19, CHK1, CHK2, DP-1, MDM2, BRCA1, BRCA2, GADD45, 14-3-3β

**Cell cycle:** CDK2, CDK4, CDK6, CDC2p34, CDC25A, CDC25B, CDC25C, Cyclin B, Cyclin D, Cyclin E, Rb

**Invasion/metastasis**: VEGF, NF-kappaB, IKK, E-cadherin, N-cadherin, HSP90, TGF-beta, osteopontin, KISS1, KAI1, uPA, uPAR, MMP9, ICAM-1, FAK, EphB2, EphB3

**Transcription factor:** p-c-Jun, ETS1, c-MYC, E2F-1, GATA, Stat1, p-Stat3, p-Stat5, p-Smad1, p-RB, PR, ERa, ERb

**Apoptosis/Autophagy:** Bax, FAS, BAD, BCL2, BID, BAK, cleaved Caspase 3, cleaved Caspase 8, cleaved Caspase 9, TRAF, p53, XIAP, NFKB, IKB, Bcl-xL, Smac, LC-3I, LC-3II, Cytochrome C, TNF, AKT1, Survivin, RIP

**Angionesis:** VEGF, VEGFR, E-cadherin, PDGF, PDGFR, TGF-beta, TGF-beta Receptor, TNF alpha, COX-2, FGF, FGFR, EPO, Ang, Endoglin, Neuropilin, MMP9

**DNA repair:** P53, ATM, Phospho-ATM (Ser1981), ATR, PCNA, BRCA1, Rad52, TDP1, ERCC1, RCA1, BTG2, CCNH, DNMT1, GADD45A, PTTG1, XRCC5

**Epithelial-to-mesenchymal transition/Adhesion**: E-cadherin, catenin, Ep-CAM, HCAM, ICAM1, VCAM1

_____________________________________________________________________________
