# Supplementary material for: Proteomics, pathway array and signaling network-based medicine in cancer
Source: Cell Div. 2009 Oct 28;4:20. doi: 10.1186/1747-1028-4-20 (PMC2780394; doi:10.1186/1747-1028-4-20)
Supplement: Additional file 4 — Effect of Cdk6 and XIAP silencing on cell viability, cell cycle distribution and necrosis. Example of functional relevance of signaling proteins. [file 1747-1028-4-20-S4.doc]

**Additional file 4, Effect of Cdk6 and XIAP silencing on cell viability, cell cycle distribution and necrosis**

|  | **Cell Viability (%)** | **Cell Cycle Distribution (%)** | | | **Necrosis (%)** |
| --- | --- | --- | --- | --- | --- |
|  |  | **(G_0_/G_1_ phase)** | **(S Phase)** | **(G_2_+M Phase)** |  |
| **Negative Control** | 100 | 66.37 | 15.9 | 17.76 | 2.14 |
| **CDK6 siRNA** | 8.26 | 83.81 | 4.6 | 10.89 | 14.46 |
| **XIAP siRNA** | 14.02 | 71.56 | 6.96 | 11.24 | 31.38 |
