# Supplementary material for: Proteomics, pathway array and signaling network-based medicine in cancer
Source: Cell Div. 2009 Oct 28;4:20. doi: 10.1186/1747-1028-4-20 (PMC2780394; doi:10.1186/1747-1028-4-20)
Supplement: Additional file 5 — The expression of signaling transduction proteins in HCCs from 4 patients. Examples of the expression level of signaling proteins in HCC. [file 1747-1028-4-20-S5.doc]

**Additional 5:  The expression** of signaling transduction proteins in HCCs from 4 patients

| **SIGNALING PROTEINS** | **PATIENT SAMPLES** | | | |
| --- | --- | --- | --- | --- |
|  | **A** | **B** | **C** | **D** |
| **Akt** | 0 | 2651 | 1724 | 1438 |
| **BRCA1** | 0 | 1778 | 0 | 0 |
| **cdk6** | 652 | 0 | 0 | 0 |
| **cPKCα** | 13879 | 462 | 269 | 2058 |
| **ERK1/2** | 0 | 2308 | 186 | 0 |
| **HIF-3α** | 0 | 885 | 0 | 0 |
| **p27** | 9104 | 702 | 8470 | 9571 |
| **XIAP** | 0 | 0 | 2036 | 10094 |
